# Augmented reality—The way forward in patient education for intracranial aneurysms? A qualitative exploration of views, expectations and preferences of patients suffering from an unruptured intracranial aneurysm regarding augmented reality in patient education

**DOI:** 10.3389/fbioe.2023.1204643

**Published:** 2023-08-04

**Authors:** Julie Urlings, Inger Abma, René Aquarius, Marlien Aalbers, Ronald Bartels, Thomas Maal, Dylan Henssen, Jeroen Boogaarts

**Affiliations:** ^1^ Department of Neurosurgery, Radboud University Medical Centre, Nijmegen, Netherlands; ^2^ 3D Lab Radboudumc, Radboud University Medical Centre, Nijmegen, Netherlands; ^3^ Department of Medical Imaging, Radboud University Medical Centre, Nijmegen, Netherlands; ^4^ IQ Healthcare, Radboud Institute of Health Sciences, Radboud University Medical Center, Nijmegen, Netherlands

**Keywords:** augmented reality, mixed reality, patient education, interview study, qualitative research

## Abstract

**Objectives:** The goal of this project is to explore the views, expectations and preferences of patients with an unruptured intracranial aneurysm regarding the use of AR in patient education.

**Methods:** To gain an in-depth understanding of the patients’ perspective, a face-to-face interview study was conducted using an interview protocol with a predefined topic list. All interviews were audio-recorded and transcribed verbatim afterwards. Transcripts were analyzed using thematic content analyses. Coding was performed using Atlas.ti software.

**Results:** Seventeen interviews were conducted. The views, expectations and preferences of patients regarding patient education with AR could be subdivided into 15 categories, which could be grouped into 4 general themes: 1) experiences with current patient education, 2) expectations of AR in patient education, 3) opportunities and limitations of AR, and 4) out-of-hospital use of an AR application. Patients’ expectations were predominantly positive regarding improving patients’ understanding of their medical situation and doctor-patient communication.

**Discusssion:** This study suggests that patients with unruptured intracranial aneurysms are open to receive patient education regarding their disease with AR. Patients expect that AR models can help patients with intra-cranial aneurysms better understand their disease, treatment options and risks. Additionally, patients expect AR could improve doctor-patient communication.

## 1 Introduction

An intracranial aneurysm is a dilation in the wall of an artery of the brain. This increases the chances of the blood vessel rupturing, which can cause severe bleeding. However, aneurysm treatment is associated with possible complications. Therefore, the risk of spontaneous rupture has to be balanced against the risks of procedural complications. Clinical decision making is therefore complicated and for patients with unruptured, often asymptomatic, intracranial aneurysms, the process of information transfer needs to be in-depth and detailed. Physicians have the responsibility to inform patients about the aneurysm itself, the different treatment options (e.g., endovascular coiling, open surgical clipping) and the associated risks. This process of clinical decision making can be challenging. Survey studies reported that there was low agreement between patients and neurosurgeons regarding the “best” treatment option for each individual patient (King et al., 2005; Saito et al., 2012). Furthermore, almost no agreement with regard to the understanding of treatment options and corresponding risks has been reported (Saito et al., 2012) (King et al., 2005). Patients estimated much higher risks of stroke or death from surgical clipping, endovascular embolization, or no intervention compared with the estimates offered by their neurosurgeons (King et al., 2005). These results illustrate that important discrepancies exist between the perceived risks and benefits as estimated by neurosurgeons and those estimated by patients. Patient education with innovative 3D visualization techniques might be able to overcome these discrepancies.

AR is a form of 3D technology which overlays a computer-generated image on a user’s view of the real world, providing additional data and context (Barsom et al., 2016). Although AR is already being studied and used in the education of students and residents (Kamphuis et al., 2014; Zhu et al., 2014; Pelargos et al., 2017), its benefits in the context of patient education are mostly unknown (Urlings et al., 2022). Theoretically, the use of AR might add to standard methods of information transfer as AR has the ability to simulate events on top of reality, creating a hybrid immersive learning environment. This could facilitate the development of skills, such as problem solving, critical thinking and communicating (Dunleavy et al., 2008). Additionally, studies on AR for anatomy education suggest that using AR applications decreases cognitive load in students (i.e., the amount of working memory resources used) (Iordache et al., 2012; Di Serio ÁIbáñez and Kloos, 2013; Kucuk et al., 2016; Henssen et al., 2020). In a conference paper of Jakl et al. (2020) participants thought that an AR system as a complementary tool for medical patient education could lead to an improved understanding of the content of a medical consultation. Considering these benefits of AR for students and residents, we hypothesize that AR could also be beneficial for patient education.

However, before implementing AR in the education of unruptured intracranial aneurysm patients, it is important to ask these patients if and how they would like to see AR used in the clinic. Patients’ views on the current patient education process and their expectancies of the usage of AR, can provide valuable insights into effective AR implementation. Previous work has shown that including patients through qualitative research can result in identifying facilitators and barriers from the patient perspective, and positive and negative effects of new educational tool usage (van de Belt et al., 2018; Hilt et al., 2020).

This qualitative study aimed to provide more insight in the expectations and wishes of patients suffering from unruptured intracranial aneurysms on AR in patient education. These insights could provide indications for AR in patient education, and thus form a basis for developing a suitable AR application and for further research.

## 2 Materials and methods

### 2.1 Study design

To obtain an in-depth understanding of the subject and to explore patients’ views, expectations and preferences, a qualitative research design was used. Ethical approval was not required for this type of study under Dutch law, and an exemption was obtained by the local Medical Ethics Committee “CMO Regio Arnhem-Nijmegen” (registration number: 2020-7206). Written informed consent was obtained from all participants. Data were collected using semi structured interviews to obtain a nuanced understanding of the patients’ expectations and wishes concerning AR in patient education.

Based on a literature study, an interview protocol was constructed based on a topic list. In collaboration with a radiologist and neurosurgeons and 3D technicians, the topic list was finetuned. The interview protocol provided structure for the interview to ensure that all necessary topics were covered. Interviews were conducted from May 2021 until December 2021.

The interviews were conducted by two trained researchers (JU and/or D.H.). Each participant was interviewed once, at a time and place suitable for the patient. The setting of this interview was informal and was conducted as a conversation. Based on patients’ preferences, close relatives attended the interview and were allowed to assist patients during the interviews. The researchers made explicit that they had no involvement in the medical care of the participants.

Interviews were conducted face-tot-face or by use of video call programs due to the COVID-19 pandemic and its subsequent restrictions (e.g., social distancing). All interviews were audio-recorded and transcribed verbatim after conducting the interview. Additional interviews were conducted until data saturation was suspected (i.e., no new topics emerged during the interviews), after which two additional interviews were held to confirm this.

Open‐ended questions were used as starting points for the interview. The first part of the interview consisted of three topics: 1) general/demographics, 2) experiences with current patient education, and 3) context (friends and family). Then, to clearly illustrate augmented reality technology to the participants, two videos were shown during the interviews. These videos can be viewed in Supplementary Material SA, B. The first video gave a general idea on what AR looks like. The second video showed an AR aneurysm model. The interview guide used after watching the first video consisted of the topic 1) first reaction to AR. After watching the second video, the guide consisted of six major topics: 1) first reactions to AR, 2) expected value of using 3D prints during consultation, 3) wishes for AR, 4) the AR model, 5) context (friend and family), 6) additional experiences and needs. The main topics/questions are shown in Table 1. Patients were encouraged to express their own opinions and experiences freely. Clarification was asked regularly to ensure that answers given were understood correctly. When new topics emerged from the interviews, they were added to the topic list.

**TABLE 1:** Timeline of each interview including main interview topics and accompanying interview questions.

Interview topics	Main questions
General/demographics	- Questions on age, work and occupational status
	- Can you tell me about your aneurysm?
	- How were you diagnosed?
	- Which treatment did you receive?
Experiences with current patient education	- How did your physician educate you about your aneurysm?
	- What was your opinion about the information you received from your physician?
	- Did your physician use visualization tools?
	- Did you fully understand the information you received from your physician?
	- What was the most important information you received from your physician?
Context (family and friends)	- Did you talk about your condition with family and/or friends?
First video is shown	
First reaction to AR	- What are your first reactions on AR after watching this video?
	- Did you already have experience with AR?
	- Do you have any questions after watching the first video?
Second video is shown	
First reaction to AR	- What are your first reactions on AR after watching this video?
Expected value of using 3D prints during consultation	- Do you think there are advantages of using AR in patient education?
	- Do you think there are disadvantages of using AR in patient education?
	- Do you think AR could change something about the subjects that are being discussed in the consultation room?
Wishes for AR	- If you look back on the education that you received, do you think you would have liked the education to have been with the use of AR?
	- Besides the features of AR that you saw in the video, are there other things you would like to see in AR?
	- When and where would you like to use AR?
	- On which devise would you like to see AR?
The AR model	- What did you think of the AR model that was used in the video?
Context (friend and family)	- Do you think AR could help you talk about your condition with family and friends?
Additional experiences and needs	- Do you have additional topics you want to discuss?
	- Do you have any questions?

### 2.2 Participants and recruitment

Patients were eligible for inclusion if they had a diagnosis for an intracranial aneurysm or they were already treated for their intracranial aneurysm. Patients were identified through the outpatient clinic of the Radboud university medical center, Nijmegen, Netherlands, or the weekly neurovascular interdisciplinary meeting of the first 3 months of 2021 of this hospital. Patients were first approached by a neurosurgeon or nurse practitioner. If patients were interested to participate in this project, their contact details were passed on to the researchers who then provided more information on the study. To seek in‐depth information from a wide range of patients, purposive sampling was conducted based on gender, age and educational background. Only patients with a sufficient understanding of the Dutch language were included in the study.

### 2.3 Data analysis

The data from these interviews were analysed using thematic content analysis. This was done using Atlas. ti software version 22 Windows (http://atlasti.com; ATLAS.ti Scientific Software Development GmbH, Berlin, Germany). The first interviews were independently coded by two authors (JU and DH). The assigned codes were compared and discussed until consensus was reached on the codes for the codebook. Analysis took place via an inductive iterative process using the constant comparative method. This means that data analysis started after the first interview was completed. As the study continued, codes derived from the previous interview(s) were used as a starting point for coding the next interview, adding additional codes where and whenever needed.

JU and DH also started axial coding in which codes were linked together and combined into categories. This process continued throughout the coding of the subsequent interviews, in which JU analysed the interviews first, adding new codes if needed, after which DH checked the coded interviews for agreement. Together, they adapted the codebook throughout this process and created an overview of the code categories and themes. Finally, the findings were discussed with IA and JB, and the final categories and themes were decided upon.

## 3 Results

### 3.1 Patient characteristics

Seventeen patients with an unruptured intracranial aneurysm were interviewed. Patient characteristics are summarized in Table 2.

**TABLE 2:** Patient characteristics.

	Participants (Brown et al., 2019)
Gender	
- Female (n)	11
- Male (n)	6
Median age	65 years (20–75 years)
Occupational status	
- Working (n)	9
- Unable to work (n)	5
- Retired (N)	3
Treatment	
- Minimal invasive (Coiling/surpass flow diverter)	10
- Clipping	1
- Watchfull waiting	5
- Clipping and watchfull waiting	1
Level of education	
- Bachelor’s degree	7
- No Bachelor’s degree	10
	

### 3.2 Results of the analysis

The patients’ expectations and wishes on AR were categorized into 15 categories. These were sorted into 4 themes: 1) experiences with current patient education, 2) expectations of AR in patient education, 3) opportunities and limitations of AR, and 4) out of hospital use of an AR application. Details are summerized in Table 3.

**TABLE 3:** Themes and categories.

Themes	Categories
Experiences with current patient education	- Received education
	- Asking questions
	- Visualization tools
	- Points of improvement
Expectations of AR in patient education	- Improving patients’ understanding
	- Emotional confrontation
Opportunities and limitations of AR	- Wishes used AR model
	- Explaining relate complaints
	- Showing treatment options in AR
	- Patients’ needs
	- Necessity
	- Concerns regarding the use of AR
	- Timing
Out- of- hospital use	- AR at home
	- AR for relatives

#### 3.2.1 Current patient education

This theme describes experiences patients have with current patient education, which can provide valuable insights into effective AR implementation.

##### 3.2.1.1 Experiences

Participants mentioned that creating clarity and providing assurance was the most important goal of patient education. They indicated that this could be achieved by providing complete, though concise and understandable information. Additionally, it was mentioned that it was very important for patients to be able to trust their physician and that the education is approached positively, i.e., not just focusing on the risks. This is important because emphasizing the risks of an intracranial aneurysm could cause patients to feel overly anxious.

When discussing which information, they valued the most, interviewees reported that detailed information about aneurysm location, size and origin was important to them. The participants described they needed this information to understand the explanation of different treatment options, the advantages and disadvantages, the associated risks and the possible consequences of rupture or treatment failure.

It was expressed by participants that they had forgotten a great amount of information that was given during the first consultation. The reasons that people gave for this memory loss were pre-existent memory problems, being nervous and/or the excessive amount of information given during their first visit. Interviewees told us that they therefore learned most about their aneurysm by reading information provided by their physician or information they found online.

Participants stated that they found it difficult to formulate the right questions during the first consultation due to shock and the amount of information provided at this first consultation. Therefore, most questions arose after their hospital visit.

#####  3.2.1.2. Visualization tools

Two tools were described by the interviewees which were used to help them visualize the aneurysm: radiological data and a sketch made by the physician. A mentioned disadvantage of the radiological data was that participants found it too difficult to understand. They mentioned that they needed a lot of explanation from their physician to fully comprehend what they saw when looking at scans. One patient even stated that imaging was interesting for physicians, but not for patients.

“A neurologist explained to me that there were at least two aneurysms in there and she also showed them on a scan but well, all you see are a few gray spots. You can't do much with that. (P14)”

Patients expressed that they were more satisfied with the sketches made by the physician. The reasons patients gave for this preference was that these sketches gave them more insight in their diagnosis and possibilities for treatment in a simple way. Additionally, the physician showing a stent when explaining treatment options was well appreciated by patients.

“They also showed it on the MRI or CT, but that is less clear. It is more difficult to understand. With a sketch, it was just a blood vessel with a ball on top and then he drew some coils in it. (P4)”

#### 3.2.2 Expectations regarding AR

##### 3.2.2.1 Improving patients’ understanding

The biggest benefit patients expected from AR was that it could increase patients’ understanding of the location and size of their aneurysm. In addition, patients believed AR could be a valuable addition to help explain treatment options and the associated risks. They expected that, especially the attractive visualization used in AR would help patients better understand the information given by their physician. This might also make the information easier to remember. Moreover, patients expected that increased understanding could also lead to patients making more well-educated decisions on their treatment. Participants believed this visualization and the increased understanding that could come with it would make it easier to live at ease with their intracranial aneurysm.

“Then I can see what they’re really talking about. Because I try to understand what they are telling me, but if you really just have a clear image, it's just a lot easier. Then it becomes easier to remember and you can also explain it to others more easily. (P9)”

It was also expressed that seeing an aneurysm in AR would give more insight in possible complaints due to their aneurysm.

‘Very clear, nice to see. You understand it a little better. I suppose that if I could see this of my own head, then I would know precisely where my aneurysm is located. So, when I feel pain elsewhere, I don’t have to be afraid. But then, if I feel pain on the location of the aneurysm, I’d think, oh, could there be something wrong there? (P10)”

With regard to AR in the consultation room, views were that the use of AR would be suitable in a consultation with their physician. Patients differed in their view whether using AR would lead to discussing extra topics during a consultation. One view was that the use of AR would not change what is being discussed in the consultation room. The contrasting view was that, because patients can see exactly what the physician is talking about, AR could lead to patients asking more questions.

##### 3.2.2.2 Emotional confrontation

Before viewing the video of the AR aneurysm model, patients expected that viewing their aneurysm in AR could be emotionally confronting and even scary. However, after watching the AR video, none of the participants expressed feelings of this kind. Patients did not find the AR model scary because it was clear and informative and because it did not look too ‘real’. Having a good doctor-patient relationship was expected to make it less scary to watch the AR model, emphasizing the guiding role of the physician.

#### 3.2.3 Opportunities and limitations AR

##### 3.2.3.1 Wishes regarding the used AR model

Generally, patients thought the AR aneurysm model used in this study was clear and that it gave insight in the anatomy and the location of the aneurysm. Participants were happy with an innovation like this in patient education and mentioned they would like to see a personalized AR model. The adaptability of AR was seen as an advantage. Unlike with a sketch, with AR you can easily remove and add different layers of the model, which provides patients with a more detailed explanation. However, several points for improvement and wishes for AR emerged during the interviews. Concerning the current model, patients stated that the aneurysm in the model should be presented larger or more striking. Thereby, it was stated that more details should be incorporated in the AR model, such as cranial nerves, white matter and the thickness of blood vessels. Additionally, it was suggested that a brain should be incorporated in the AR model to make it clearer where the blood vessels are in relation to the brain. Also indicating the front and back of the model could be helpful.

##### 3.2.3.2 Explaining related complaints

When receiving information using AR, patients stated that the model should be used to explain potential causes of the aneurysm and causes of complaints provoked by their aneurysm or perceived treatment. One patient stated that they would like their physician to use AR every consult to update them on the state of their aneurysm.

##### 3.2.3.3 Showing treatment options with AR

*Participants* stated that they would like to see their treatment options depicted in AR. Some patients stated they would like AR to be used to create more insight in treatment risks, whereas others disclosed that treatment risks should not be visualized in AR because it would be too confronting.

##### 3.2.3.4 Concerns regarding the use of AR

Concerns were expressed regarding the accuracy of AR. Participants were afraid details would get lost when transferring data from a scan to an AR model or that the model will show a distorted reality.

Another problem that was raised was that patients need time to get used to the new technology, which could be difficult at an older age. Proper guidance when using AR and a physician well-trained in using the device were therefore considered necessary by patients. Additionally, patients specifically stated that when using AR, medical education provided by a specialist will always stay necessary, preferably by a physician.

“My physician could explain it in great detail with a drawing, or at least well enough, and that gave a soothing feeling. You know that the physician understands everything about it and that he will fix it. That feeling should stay if you’re going to do it that way. (P4)”

Views on preferred AR device were divided. One view was that using a phone to view AR would make is easy to watch AR at home and prevent having to purchase a new device. Several patients were inconclusive about which AR device they would prefer, because they did not have any experience in using AR. When discussing the potential disadvantages of AR in patient education, patients stated that although they would like education using AR, they were afraid it would not work in practice, because it would consume too much time during the consultation.

##### 3.2.3.5 Patients’ needs

Several perspectives on patients’ need for AR arose during the interviews. Patients told us they expected that it depends on the patient’s coping strategy whether someone wants education with AR or not. For example, some patients just do not want to know everything about their condition. It was suspected that AR might be too confronting when a patient has a more severe diagnosis or needs to undergo a treatment with more risks. It was specifically expressed that therefore you should always ask the patient whether they would like to see an AR model. Additionally, it was stated that AR would be most favorable for patients with a low understanding of their condition.

##### 3.2.3.6 Necessity

The necessity of AR in patient education was questioned during the interviews. Participants reported that AR gave them more insight in their condition, but that it was not necessary for patient education. Additionally, it was stated that AR would be more beneficial for conditions that are more difficult to understand than having an aneurysm.

##### 3.2.3.7 Timing

Participants advised not to provide AR when a patient first hears about having an aneurysm, but at a later stage. This way, patients would have time to let the news sink in before further patient education takes place with AR. However, not all patients agreed: one patient stated that he would not mind receiving AR patient education in the acute moment.

#### 3.2.4 Out of hospital use

##### 3.2.4.1 AR at home

Several patients mentioned that they would like to be able to watch an AR model of their aneurysm at home, for example, by using an AR-application on a phone or tablet. This would allow them to rewatch information in a comfortable environment with relatives. One patient expressed that they wanted to gather as much information at home as possible. A suggestion that was made by participants was that an AR recording at home should include the physician’s explanation that they received at their hospital visit.

##### 3.2.4.2 AR for relatives

AR could also be used for relatives. A reason patients mentioned to do this was that using AR could make talking about aneurysms easier for patients. Another reason was that it would be beneficial that one would not have to explain everything repeatedly, because they could instead show their relatives using AR. Reasons mentioned against using AR for relatives were that AR could make relatives uncomfortable or that relatives would not be interested in AR.

## 4 Discussion and conclusion

### 4.1 Discussion

This study explored the views, expectations and preferences with regard to the use of AR in educating patients about their intracranial aneurysms. In general, participants expressed that AR could be useful in this setting, particularly with regard to improving patients’ understanding of their medical situation. Also, AR was suggested to improve the communication between doctor and patient. It was preferred to use AR later in the educational process (i.e., not directly after receiving the diagnosis) and most interviewees saw opportunities regarding the use of AR at home and in explaining their disease to their relatives. Nevertheless, interviewees disclosed that AR could be too time-consuming during consultation and that a physician trained in using AR is necessary. Also, AR was believed to be emotionally confronting, although none of the participants experienced this after viewing the here described AR system.

The few previous studies that have been carried out on the use of AR in patient education, rarely involved patients in the process of designing and implementing AR applications (Domhardt et al., 2015; Calle-Bustos et al., 2017; Azman et al., 2019; Brown et al., 2019; Calle-Bustos et al., 2019; Wake et al., 2019; Bray et al., 2020; House et al., 2020; Sezer et al., 2020; Tait et al., 2020). For a recent review, see (Urlings et al., 2022). End-users often stress misalignments among their problems and the solutions that technology systems aim to solve (Calvillo-Arbizu et al., 2019). Including patient views early on in the process of design of patient education is important, because physicians and other healthcare workers might not always be able to judge what is important to patients (Hilt et al., 2020). Our current study is the first research that has been performed to explore the views of patients with unruptured intracranial aneurysms regarding AR for educational purposes.

The previous studies comprised patients suffering from a diverse spectrum of chronic diseases (e.g., prostate cancer, diabetes mellitus, multiple sclerosis, epilepsy). These studies showed benefits of AR such as (perceived) knowledge gain and increased patient satisfaction (Urlings et al., 2022). These benefits are similar to the benefits of AR as suspected by the patients interviewed in our study. More high-quality studies are needed to conclude whether AR truly has those beneficial effects on patient education and whether it could thereby improve doctor-patient communication.

Additionally, inter-individual differences in visuospatial abilities significantly impacted student performance when working with AR (Moro et al., 2020).

Furthermore, it was noted that patient-specific factors, such as conditions like strabismus that affect three-dimensional vision, could affect the choice of the most suitable AR device for patient education (Jakl et al., 2020). Nevertheless, whether spatial abilities and other co-variates play a significant role when working with AR in patient education remains elusive and should be investigated in future studies.

Patients in this study emphasized the ongoing necessity of medical education provided by a specialist, preferably a physician, when augmented reality (AR) is utilized. This finding corresponds with the existing literature on the use of visualization tools for patient education. Previous research on alternative methods such as videotaped instructions or computer-aided information systems has produced mixed results. However, existing evidence shows that providing a combination of spoken and written or visual information is best (Thomson et al., 2001; Kessels, 2003).

Furthermore, patients expressed their preference to utilize AR later in the educational process as this would give patients the time to process their diagnosis before further patient education takes place. We know evidence exists, indicating that attentional narrowing occurs when events are perceived as stressful or emotional (Kessels, 2003). In such situations, the central message, such as “you have an aneurysm in your brain,” becomes the primary focus, leading to limited attention towards other provided information. Consequently, any remaining information, perhaps about treatment options and risks, is not processed and stored into memory and therefore cannot be recalled. Considering this, providing AR patient education later in the educational process could me more beneficial (Kessels, 2003).

Similar to the expectations of patients in our study that AR could help in communicating with relatives, it was found that an AR intervention helped parents to talk about a planned procedure with their children and that it significantly decreased anxiety in parents whose children were undergoing invasive procedures (Bray et al., 2020). In another study relatives expressed their preference of an AR application over a physical model and chose it as the future standard tool for patient education (House et al., 2020). The latter contrasts with our finding that some patients feared AR could make relatives uncomfortable or that relatives would not be interested in AR. Future research should examine the exact value of using AR between patients and relatives.

Finally, patients in our study expressed their worries about needing time to get used to the new technology and that this could be difficult at an older age. Proper guidance when using AR and a physician well-trained in using the device were considered necessary. When looking at the existing literature however, most studies reported a high usability and likability of the AR applications used (Calle-Bustos et al., 2017; Brown et al., 2019; Calle-Bustos et al., 2019; Wake et al., 2019; Bray et al., 2020; House et al., 2020; Tait et al., 2020). These studies comprised patients with an age range between 8 and 63 years. Five of these studies offered participants training or assistance in using AR (16, 17, 21, 22, 24). Therefore, the use of AR might not be problematic for patients with an older age as long as there is proper guidance.

#### 4.1.1 Strengths and limitations

This study is the first to present the expectations and wishes concerning the use of AR for patient education among patients with unruptured intracranial aneurysms. It has been shown in other aspects of medical treatment that the patient’s expectations and wishes can be different from those of physicians (Hilt et al., 2020). The identification of positive and negative expectations and patients’ wishes as perceived by individual participants allows us to improve the use of AR models for patient education. The results form a basis for future quantitative studies on the effect of AR in patient education for these patients. In these studies, it is necessary to determine whether the use of these models truly contributes to patients’ understanding of their disease, the procedure, and risks compared with the use of 2D imaging alone. Another strength of this study is the role of the researchers as an independent party in relation to the treatment process. This enabled participants to speak openly.

To seek in‐depth information from a wide range of patients purposive sampling was conducted in this study. As a result, the participant group comprised patients with varying genders, ages, and educational backgrounds. It is worth noting that there were more female participants than male participants, which aligns with the epidemiology of unruptured intracranial aneurysms, as these are more commonly observed in females (Vlak et al., 2011; Brown and Broderick, 2014).

A limitation of this study was that all study participants were treated in the same hospital located in the Netherlands. Patients might perceive AR differently depending on the health resources in a country. This partially limits the transferability of these findings to other patient populations.

Another limitation is that we opted for videos to illustrate AR instead of an AR application or AR-glasses due to practical considerations. This made it possible to conduct the interviews online, which was necessitated by the interviews being conducted during the COVID-19 pandemic. It is possible that this resulted in a less immersive experience compared to an application or AR glasses.

### 4.2 Conclusion

This study suggests that patients with unruptured intracranial aneurysms are open to receive patient education regarding their disease with AR. Patients expect that AR models can help patients with intra-cranial aneurysms better understand their disease, treatment options and risks. Additionally, patients expect AR could improve doctor-patient communication. The views, expectations and preferences of patients identified in this study can contribute to improving information provision and communication using AR applications by providing insights into patients’ perceptions.

### 4.3 Practice implications

Future studies on AR in patient education should take these expectations and wishes into account and evaluate the extent to which the use of AR models could positively influence the quality of patient education.

## Data Availability

The original contributions presented in the study are included in the article/Supplementary Material, further inquiries can be directed to the corresponding author.
